# NIST Mechanisms for Disseminating Measurements

**DOI:** 10.6028/jres.106.012

**Published:** 2001-02-01

**Authors:** T. E. Gills, S. Dittman, J. R. Rumble, C. S. Brickenkamp, G. L. Harris, N. M. Trahey

**Affiliations:** National Institute of Standards and Technology, Gaithersburg, MD 20899-0001, USA

**Keywords:** calibrations, certified reference materials, critically evaluated data, commerce and trade, legal metrology, measurement systems, SRM®, standard reference data, traceability, weights and measures

## Abstract

The national responsibilities assigned to the National Bureau of Standards (NBS) early in the last century for providing measurement assistance and service are carried out today by the four programs that comprise the National Institute of Standards and Technology (NIST) Office of Measurement Services (OMS). They are the Calibration Program (CP), the Standard Reference Materials Program (SRMP), the Standard Reference Data Program (SRDP), and the Weights and Measures Program (W&MP). Organized when the U.S. Congress changed the NBS name to NIST, the OMS facilitates access to the measurement and standards activities of NIST laboratories and programs through the dissemination of NIST products, data, and services. A brief historical introduction followed by a perspective of pivotal measurement developments from 1901 to the present and concluding with a look to the future of NIST measurement services in the next decade of the new millennium are presented for each OMS program.

## 1. Introduction

### 1.1 Fixing Standards for the Nation—The First Century

The National Bureau of Standards (NBS), predecessor of the National Institute of Standards and Technology (NIST), began providing the Nation with measurement artifacts and instruments in 1901 when the Office of Weights and Measures (OWM) became part of the fledgling institution. Established by Congress in 1836, the OWM was, and still is, responsible for providing uniform standards of mass, length, and volume for trade as required by Article 1, Section 8 of the U.S. Constitution. This same mandate led the Bureau of Standards (as NBS was first called) to begin providing special types of “tests” designed specifically to help U.S. industries achieve measurement uniformity by realizing base quantities identified when the Treaty of the Meter was signed by the United States in 1875. Today, NIST calibrations are leveraged thousands of times over for everything from watt-hour meters to radiation dosimeters. The first NBS physical artifacts, called “Standard Samples,” were issued in 1906 for limestone and cast iron. Analyzed by Bureau chemists for composition, these certified reference materials finally made it possible for foundries to accurately control the addition of these critical ingredients during the production of steel. Today more than 1300 NIST Standard Reference Materials are certified for chemical composition, physical properties, and engineering processes. The Bureau recognized early that technical data were essential components of the quest for scientific knowledge and in the 1920s led the worldwide effort to produce the first complete set of critically evaluated data ever compiled. A later act (1968) of the U.S. Congress gave NBS the responsibility for assuring that “reliable reference data” would always be available to the Nation’s scientific and technical communities.

### 1.2 Fixing Standards for the Nation—The Second Century

The Omnibus Trade and Competitiveness Act of 1988 substantially modified the Organic Act and in so doing renamed NBS the National Institute of Standards and Technology (NIST). The changes provided the impetus for the establishment of a new operating unit called Technology Services (TS) whose main purpose was to “…help improve the use of technology….”^1^ Within TS, the Office of Measurement Services (OMS) became the organizational umbrella for weights and measures guidance and the three services cited in the Law, namely Standard Reference Materials, Standard Reference Data, and calibrations.

The Calibration Program (CP), the Standard Reference Materials Program (SRMP), and the Weights and Measures Program (W&MP) make up the largest integrated national measurement transfer system in the world. The Standard Reference Data Program (SRDP) augments this system by providing scientists, engineers, and the general public with access to critically evaluated data necessary to perform state-of-the-art research and development. Through these four programs, expert legal metrology guidance and the measurement products and services developed in the NIST laboratories are disseminated to state and local governments, Federal agencies, and the industrial and the scientific communities. The OMS successfully places these traditional and discrete services within the context of the critical regulatory, market, and trade issues of today ensuring that traceability to national standards can be established at necessary levels of uncertainty and equity in domestic and international commerce can be achieved. The coordination and technological outreach activities of the four OMS technology transfer programs, CP, SRMP, SRDP, and W&MP, share a single goal; that is, to provide the measurement tools and technological assistance by which the Nation’s measurements can link to NIST. This metrologic goal is as important and relevant to the mission of NIST today as it was when the Bureau was established one hundred years ago.

## 2. Calibration Program

Here briefly reviewed are the origins of an international system of measurement units, the economic impact of that system on U.S. commerce and trade, how the units are maintained and disseminated through calibrations, the role of NIST in these processes, and future developments. Let us start with a definition of calibration and later examine the definition of traceability. According to the International Vocabulary of Basic and General Terms in Metrology (VIM)[1], a calibration is a “set of operations that establish, under specified conditions, the relationship between values of quantities indicated by a measuring instrument or measuring system, or values represented by a material measure of a reference material, and the corresponding values realized by standards.” There are two important parts to this concept:

- the standard to which a device is related, and
- the operations by which the calibration is carried out.

This discussion deals primarily with the standard and the impact of an internationally agreed upon set of standards.

### 2.1 The Impact of Calibrations Today

Physical measurements are the foundation of manufacturing, trade, and research. In today’s world, these measurements generally start with an internationally agreed upon set of standards, the values of which are transmitted through legions of laboratories and manufacturers to the plant floor and the store cash register. In the United States, NIST standards and measurements serve as the starting points for the dissemination of these values through calibrations and other services. The actual number of calibrations that NIST performs is quite small, given the scale and diversity of the U.S. economy, but their impact is substantial. This is possible because of the enormous leverage of NIST measurements. Because of the vastness of the U.S. system, the full impact of NIST’s calibration services can only be estimated. However, it is known that

- NIST calibrations of 50 sets of gauge blocks are transferred to about 26 000 sets, used in turn, to calibrate about 2.6 million blocks;
- NIST has 30 watt-hour meter customers who are responsible for the calibration of some 100 million watt-hour meters monitored by utilities measuring 2.7 trillion kilowatt hours of electricity annually and bring in $180 billion in revenues;
- NIST has performed 73 ultrasonic measurements over the last 10 years for 17 customers that support a $2.5 billion industry;
- fewer than 30 sets of masses are calibrated each year by NIST for state laboratories that support 14 500 customers with over 300 000 mass measurements for legal metrology and industry use;
- NIST thermocouples calibration services save thermocouple manufacturers an estimated $2 million annually; and
- the semiconductor industry utilizes NIST calibration services to support temperature, dimension, dose, optical properties, pressure, flow rate, contamination, radio frequency power, time, magnetic field, stress and strain, and electrical measurements costing over $2 billion per year.

These examples illustrate the leverage that a few NIST calibration measurements can have. They also indicate the importance of measurements to the U.S. economy and their traceability to national standards.

### 2.2 Organizing Early Chaos

From earliest times, humankind has developed standards for manufacture and trade. The earliest attempts to develop organized measurement systems relied on easily available artifacts and concepts, e.g., naturally occurring materials, body dimensions, a day’s work. All of these suffered from the lack of reproducibility, portability, and durability.

As trade grew and manufacturing activities increased both in volume and economic importance, the need for nationally and globally accepted standards became increasingly necessary. The ability to adopt and maintain these standards got a major boost in 1875 with the Treaty of the Meter that established measurement standards and provided a structure to review research, debate findings, and recommend and adopt new and revised units [2]. At first, the metric system had only three quantities—length, time, and mass. In 1946, the International Committee for Weights and Measures (CIPM) approved the ampere. In 1954, the ampere, candela, and kelvin were formally added to the metric system, and the final unit, the mole, was added in 1971 by the General Conference on Weights and Measures (CGPM)—the highest level body under the Treaty of the Meter. (In 1960, the metric system was formally renamed the International System of Units, universally abbreviated SI, by the CGPM.) This number of units remains unchanged to this day; only the methods of realizing them have evolved.

Even after the adoption of the metric system, it took more than 15 years for the United States to stabilize its measurement units. In 1892, a professor reported that he had a choice of any of “eight different ‘authoritative’ values for the U.S. gallon” and he had resolved the matter by using his own—a ninth [3]. The impact of this chaos was clear: “In the decade before 1900, the export of American manufactures almost doubled. Only Germany’s overseas trade had exceeded this rate of increase in the same period largely because, as our manufacturing and trade associations pointed out, [Germany] was able to guarantee the uniformity of and quality of her exported goods [4].”

Despite the relatively recent advent of the SI in 1960, the seven base quantities, and the units derived from them (e.g., for force, pressure, voltage), and some “accepted” units are now the foundation of today’s global measurement system. Even in the United States, where the SI is used along side other systems, many familiar products are packaged in common SI or SI-related units. For example, soft drinks and alcoholic beverages are sold by the liter, film is sized in millimeters, pharmaceuticals are prescribed in milligram and microgram dosages, and food packages are labeled in both SI or SI-related and customary units.

### 2.3 Capitalizing on the New System

Following the adoption of a uniform system of units, countries began authorizing the formation of national metrology institutions (NMIs), many of which were established at the turn of the 20th century. This burst of activity reflected the astonishing growth of the industrial revolution in manufacturing, use of electricity, and radio propagation among other industries and thus, the need for standards for commerce, especially physical measurement standards that were well defined, robust, and capable of being measured with appropriate accuracy.

Accepted international standards today are realized by experiments (with the exception of mass). Modern metrology has kept pace with and embraced technology to define standards for quantities. Barley corn has been replaced by frequency measurements and saturated cells have been replaced by quantum-based phenomena. In the future, one can look toward counting individual electrons to measure current and capacitance. Realizing measurement standards in these new and exciting ways allows us to achieve the required portability, reproducibility, and durability. It also provides flexibility and allows improved accuracies to be incorporated into technology. However, it also requires continuous intercomparisons among NMIs maintaining the standards to ensure that unit realizations are correct and uncertainty statements are well founded.

### 2.4 The Flow of Calibrations

**Fig. 1:**
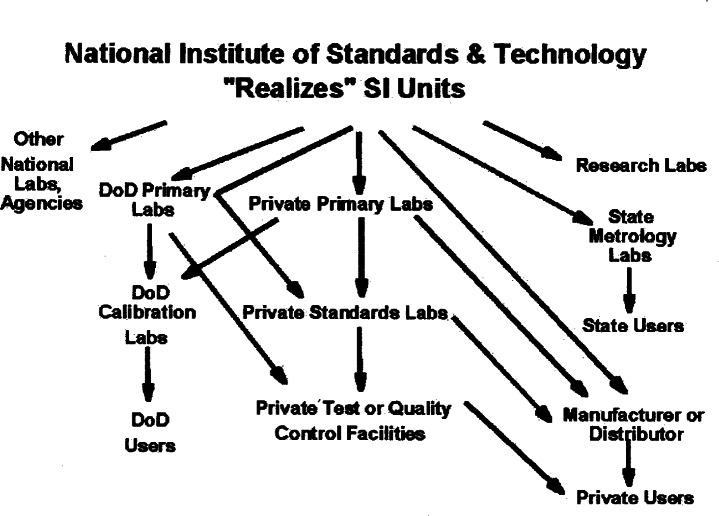
Traceability to the SI.

Let us step back and look more closely at the transmission process used to bring the SI system to the plant floor (see Fig. 1). It is one thing to have an agreed upon system of units and quite another to make sure that every type of measurement is tied to it. In the physical measurement world, this linkage is maintained through calibrations. Suitable physical measurement artifacts may be sent to an NMI to be compared against its standards. The comparison is made, and a report is prepared showing the readings of both systems. The artifact and the report are returned to the owner who may then transfer those readings (measurements) to internal or working standards that in turn, may then be transferred to other customers (government or private), the plant floor, and specific products. Ultimately, these measurements may be tied to international standards over long and varied pathways.

In the United States, NIST serves as the dissemination point for the base SI and derived units. The job of NIST is twofold; to ensure U.S. national standards are accurate realizations of the SI units and to transfer the values of those standards to the U.S. measurement system through calibrations and other types of measurement services. By sending artifacts and standards to NIST or to customers of NIST services, a U.S. user can achieve linkage to the SI system maintained by nearly 50 countries around the world. In this manner, the ability to make components at manufacturing facilities around the world and to trade them across national boundaries and to compare research results with colleagues in different countries, become realities.

### 2.5 When is a Calibration Traceable?

**Fig. 2:**
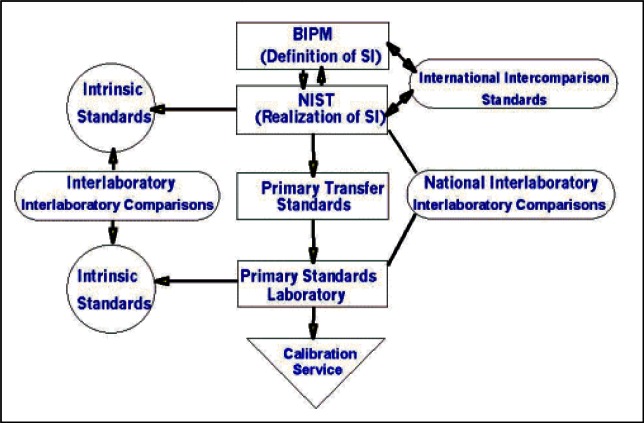
Maintaining equivalence of standards (BIPM: International Bureau of Weights and Measures).

With an appropriate system of units and NMIs in place, traceability becomes a concept that could be well-defined and achievable (see Fig. 2). Let us first examine what traceability is and what is not.

The definition of traceability that has achieved global acceptance in the metrology community is as follows:

“… the property of the result of a measurement or the value of a standard whereby it can be related to stated references, usually national or international standards, through an unbroken chain of comparisons all having stated uncertainties.” [5]

It is important to note that traceability is the property of the result of a measurement, not of an instrument or calibration report or laboratory. It is not achieved by following a special procedure or using special equipment. This underscores that merely having an instrument calibrated, even by NIST, is not enough to make it traceable to the appropriate SI unit. The measurement system by which values are transferred must be clearly understood and under control.

**Fig. 3:**
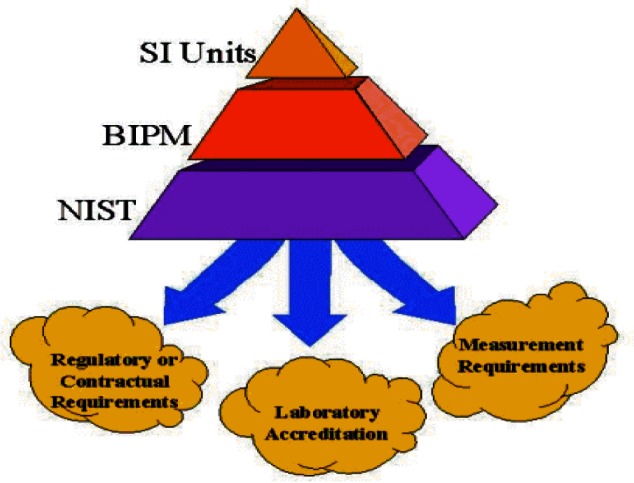
U.S. calibration hierarchy.

Our understanding of traceability has matured from merely following a paper trail to understanding that an underlying measurement system must be in place. As shown in Fig. 3, the approach today relies on sound quality system principles, assessment or accreditation processes, and documented uncertainties. These components are incorporated in the definition when it speaks of “*an unbroken chain of comparisons all having stated uncertainties*.”

The definition of traceability refers to stated references. These may be SI units, derived units, consensus standards, corporate in-house units, etc. In the U.S., the most common use of the term is “traceable to NIST.” While this may be what the customer intends, one should remember that being “traceable to NIST” really means being traceable to the SI. This distinction is important especially when measurements flow across national borders. The questions a purchaser of measurements performed in another country must now ask are the following: “Is the agreement between the NMIs that must support these measurements suitable to my purpose;” and, “How well are measurements propagated within each country from the supplier to the customer?”

With agreed-upon physical standards and the introduction of internationally accepted guides and standards for assessing calibration laboratories^2^,^3^, a network of accredited calibration laboratories has been developed around the world. Accredited laboratories meet relevant parts of the ISO 9000 quality series^4^ and must demonstrate that they maintain the technical competence to make specified measurements over specified ranges to specified uncertainties. An important part of accreditation is the second- or third-party verification of the traceability of a laboratory’s measurements to an appropriate standard. Knowledge and confidence that these three aspects of a measurement are under control is important for customers, especially when first-hand verification is not possible as is often true in international commerce.

### 2.6 History of the NIST Role

While NIST’s mission reaches far beyond “fixing the standards of weights and measures throughout the United States,” that role is the focus of this discussion. NIST realizes the base units and provides access to these SI units for U.S. industry, government agencies (local, State, Federal), and academia. This duty was spelled out in the Articles of Confederation and the U.S. Constitution and has been developed and clarified through a series of Congressional actions and laws.

The National Bureau of Standards (now NIST) was established in 1901 and significant organizational changes were made to it in 1988. However, its mandate for maintaining the national standards is still clear: “… to develop, maintain, and retain custody of the national standards of measurement, and provide the means and methods for making measurements consistent with those standards; …to assure the compatibility of United States national measurement standards with those of other nations.” [15 U.S.C. 271]

The role of maintaining national measurement standards includes providing access to those standards. Over 800 companies each year take advantage of the opportunity to tie their internal measurement standards to NIST standards and hence, to the SI units. Those companies, in turn, use their standards to provide calibration services to their customers, to meet regulatory requirements, and to provide quality assurance in their manufacturing processes.

NIST also supports laboratories that provide calibrations through its National Voluntary Laboratory Accreditation Program (NVLAP), that began accrediting calibration laboratories in 1994. While the concept of accredited calibration laboratories is relatively new in the United States, it permits U.S. customers to purchase calibration services with greater confidence, and it helps overseas customers feel confident that U.S. laboratories are held to the same criteria that their own laboratories must meet in order to be accredited.

### 2.7 History of the Calibration Program

At one time, NBS performed considerable numbers of measurements for other government agencies such as the testing of tires and cement, the income from which grew to $5.4 million in 1963 ($29 million in 1999 dollars). A conscious decision was made to stop testing work and concentrate on the more demanding calibrations. NBS/NIST has a history of transferring mature calibration capabilities to private industry. This allows the agency to transfer resources into meeting new measurement needs while making sure that mature measurements are continuously available for U.S. industry. These transfers have not always been successful for economic reasons but, for the most part, have worked well. Successful examples include color, viscosity, ionizing radiation, and gear-part measurements.

As the U.S. economy grew and became more sophisticated, more and more companies and federal agencies came to NBS for measurement services. Programs were established to deliver services in a somewhat *ad hoc* way. The NBS Calibration Program (CP) was established in 1967 to provide coordination, across NBS, of the technical groups that provide calibrations and to handle the administration of purchase orders and paper work. Today CP provides a central inquiry point for customers, handles customer complaints and billing problems, brings together customers and NIST service providers, represents NIST at technical meetings, facilitates communications between NIST technical groups, and provides policy support and interpretation. Future plans include working with groups throughout NIST to reengineer business processes, create a virtual library for calibration customers, benchmark measurement service delivery at sister NMIs, facilitate strategic planning for measurement services, and implement new strategic partnerships to construct a stronger U.S. national measurement system.

### 2.8 The Calibrations of the Future

Many exciting possibilities lie in the future. As already mentioned, the use of measurement systems rather than artifacts to realize the SI units has allowed metrologists to make use of technological and research advances to improve measurement standards. Some examples are given below.

- *Quantum-based standards*—The advent of quantum-based standards has brought uncertainties down to undreamed-of levels and has transferred the highest possible accuracies directly to the user. It is now possible to perform NMI-level realizations of voltage and resistance in a commercial laboratory. Of course, these realizations require all the care, training, and intercomparisons of a NMI-based realization.
- *Remote calibrations*—At the present time, only time and frequency can be disseminated by remote broadcast. This has allowed the world to achieve unparalleled accuracy in navigation. Others units are not yet realized in ways that permit remote transmission. However, researchers are looking for ways to incorporate new information technologies into metrology.
- *One-time calibrations*—Of considerable interest in some areas are standards that require a single, initial calibration. They have inherent characteristics that make them extraordinarily stable over time. An example of this is the gold/platinum thermocouple. Of course, the user must still maintain a quality system to verify that the thermocouple is working properly.
- *Non-artifact based standards*—Mass is the last measurement standard that is defined in terms of an actual artifact. Research is being conducted by several NMIs around the world to develop a non-artifact based realization of the kilogram. Two pathways that are being explored are relating mass to the Planck constant and relating mass to the mass of silicon atoms.
- *Specific traceabilities, not just to the SI*—In some areas of metrology, industry is demanding traceability to specific items, not just the pertinent SI units. An example of this is dimensional metrology where laboratories must now demonstrate traceability to a set of specific geometric conditions, not just to the meter. This demand can stress the resources of NMIs, especially in technology-driven economies like the United States, and should encourage the growth of secondary laboratories.
- *Accreditation*—In addition to the assessment of traceability to SI base and derived units, accreditation criteria are being developed in non-traditional fields such as reference material providers for water-quality testing facilities. As global trade continues to expand and as companies seek to reduce the burden of multiple customer audits, we expect accreditation of calibration and testing laboratories to increase. Additional drivers include the ISO 9000 quality series, the automotive industry’s QS 9000 quality standard, and the aircraft industry’s AS 9000 quality standard.
- *International activities*—The international metrology community has begun a major effort to provide the data that customers around the world need to acquire measurements and measurement equipment with confidence. The approach is multi-pronged including intercomparisons of measurement standards, evaluations of accreditation programs, and the preparation of an international database to help customers decide if the degree of equivalency between and among NMIs is sufficient to support the use of measurements from laboratories not located in their own countries. At this time many of the regional accreditation bodies have signed the CIPM-sponsored Mutual Recognition Arrangement (MRA) and a system of intercomparisons among NMIs has been established under the auspices of the BIPM. For the latter, two databases have been designed. One database will document the results of international comparisons between and among NMIs; the other database will document NMI calibration capabilities. More information about these databases is available at http://www.bipm.fr and http://icdb.nist.gov.

### 2.9 The Future of NIST Calibration Services

NIST has always been justly proud of the individual attention each of its calibration customers receives by the many technical and administrative service providers throughout NIST. The individualized and customized NIST service provided at the highest accuracies available in the Nation naturally comes at a high price both in dollars and time.

In addition to the technological possibilities that lie before us, there are special opportunities to improve the provision of NIST measurement services using advances in information technology. Via the Internet, NIST already provides information about the status of customer calibrations and we envision that NIST will soon be able to offer customers the ability to place orders over the Internet and obtain secure calibration reports.

In order to track and manage its workload more effectively, NIST has automated [using a work tracking and reporting system called the “Information System to Support Calibrations (ISSC)”] and integrated many steps in its calibration processes, especially those requiring handoffs and quality checks. These efforts are resulting in significant decreases in turnaround time and fewer instances of failure to meet promised calibration dates.

As part of its commitment to facilitate international trade, NIST has adopted a quality policy that will ensure the acceptance of its calibration capabilities into the CIPM MRA Appendix C. The Calibration Program can envision an even stronger quality system for NIST as an institution that not only ensures the continuity of its measurement services, but also pledges it will maintain the resource levels necessary to serve U.S. industry.

The demands on NIST for increasingly complex and rigorous measurements always outstrip its resources. CP envisions a stronger national metrology system that makes use of the excellent secondary laboratories in the U.S. Through accreditation and through partnerships, NIST can bring the highest accuracies closer to the end user while expanding the variety of available services. Such a system would ultimately allow users to purchase measurements with confidence from the most suitable source.

A limited amount of training has always been provided by NIST at the Measurement Science Conference (MSC) and at NCSL International annual meetings. To provide more training by NIST when and where it is needed, CP envisions use of the Internet for remote training and consultations and issuing instructional training courses on compact disks (CDs). A pilot program is already underway with 11 state laboratories. In addition, CP is investigating use of the Internet to help NIST expand it capabilities to “seize” control of remote facilities to augment calibration data or to actually perform calibrations. Other tools of the information age will enable NIST to expand its calibration services in ways that were not possible a few years ago. The result will be a repertoire of services that extends well beyond traditional calibration processes. There is no limit to where NIST and its customers can go together.

### 2.10 Summary

Looking to the future, we are reminded about the statement long ago that the Patent and Trademark Office should be closed because all the inventions that could possibly be invented had already been invented. The only limit to improvements in physical measurement standards is the imagination of today’s metrologists. As technical demands increase, as new industries appear, and as new technical knowledge is gained, metrologists will be called on to even better describe the base and derived units constituting the SI, both in terms of their definitions and of their realizations. In turn, this will allow improved transfer standards and sensors to be developed. NIST is proud to be at the forefront of much of this research and to be an active partner with other NMIs in the measurement developments facilitating international trade.

## 3. Standard Reference Materials Program

NBS/NIST has certified and issued several thousand different kinds of Standard Samples and Standard Reference Materials (SRMs) over the course of its first 100 years. SRMs are well-characterized homogeneous materials or simple artifacts with specific chemical or physical properties certified by NIST. They are widely used throughout the United States and the world to help develop test methods of proven accuracy, to calibrate measurement systems used to maintain quality control of the production of materials and goods, and to assure the long-term reliability and integrity of measurement processes. Their use contributes to equity and productivity in commerce and industry. They also lead to advancements in this country’s efforts to improve the quality of the environment and to preserve and improve human health. The following section describes how NIST SRMs have become hallmarks of excellence and acceptability that are international in scope and metrologic impact.

### 3.1 The First Standard Samples

In 1906, 5 years after the U.S. Congress established NBS, the research and measurement standards laboratory produced and issued the first samples of materials for standardizing analytical techniques and methods. Today the NIST inventory of standard samples issued under the trademark, SRMs^®^^5^, consists of over 230 000 units of some 1300 different products. Each year nearly 34 000 units are sold to over 7000 customers, 34 % of which are located outside the U.S.

Why did NBS begin and NIST continue in this role? In 1905, the American Foundrymen’s Association (AFA)^6^ approached the Bureau with a serious problem. The iron and steel industry was greatly concerned that foundries throughout the country lacked the necessary standards to assure the production of cast iron of uniform quality. (Note—the term “cast iron” identifies a large family of ferrous alloys that are used in the automotive industry for cylinder blocks, cylinder heads, pistons, camshafts, clutch plates, oil pumps bodies, transmission cases, gear boxes, clutch housings, and brake drums. The family of cast irons now extends far beyond the automotive industry, as the U.S. cast iron industry currently does $12 billion worth of business per year.)

Authorized under its Organic Act to carry out research “in determining the properties of materials,” the Bureau viewed the AFA request as an opportunity to produce reference materials as one means of assuring fulfillment of its mission to provide tools for achieving measurement reliability. Thus, the Bureau responded to its first industry request by producing the first seven Standard Samples, four of which were cast irons. One of these early Standard Samples, SRM^®^ 5m, is in its 16th renewal, ushering in the 90th year of availability of this benchmark standard.

Each of the 1300 existing SRMs is the result of collaboration between NIST and representatives of science and industry. The latter are customers who, for the last 100 years, have come to depend on NBS/NIST to supply the reference materials they need for accurate and reliable measurements that link to a national measurement system with SRMs serving as crucial reference points. Consequently, the use of SRMs for measurement reliability contributes to the strength of the U.S. economy and the well-being of its citizens. In recent years, other countries and international organizations have begun to cooperate with NIST in reference materials activities as they seek to capture similar benefits for their own constituencies. In its second 100 years, NIST will continue to proceed according to tradition and mission directive to meet the increasing measurement needs of a strong national economy through a responsive and effective SRM Program.

### 3.2 The Early Decades

Testing is a means to and a measure of success. Reduce the time and effort it takes “to get it right” and productivity increases. That is what the NBS/NIST Standard Samples and SRMs have been about for the last 94 years. They are some of the best illustrations of the NBS/NIST unwavering commitment to provide the necessary tools for improving productivity through valid laboratory measurements. As long as a strong industry need exists for a SRM and it is not available from another source, NIST will to continue to issue it.

While the American Foundrymen’s Association was the first to approach the Bureau with such a need, others quickly followed. By 1911, at the request of such associations and organizations as the American Steel Manufacturers and the American Chemical Society, NBS had issued 25 different Standard Samples. Included were materials for determining the composition of steels, brasses, ores, sugars, and a combustion sample for calorimetry. Materials research and analytical method development conducted by NBS/NIST have always been the central resources for its reference material activities. For many years, the NBS/NIST analytical chemistry program served almost exclusively as the basis for SRM development. Today, however, virtually all NIST technical areas, from radiation research to the engineering sciences, participate in SRM development and production.

#### 3.2.1 From Standard Samples to SRMs

Just as NIST research and development studies fuel the current SRM Program, the production of NBS Standard Samples gave rise to technical advances. One striking example occurred during World War I. Before the war, the Bureau issued a sugar Standard Sample^7^ for three important applications: standardizing saccharimeters, measuring the heat content of fuels, and differentiating bacteria in medical laboratories. Germany had been the source of the pure sugar used for characterization and certification but when deprived of this source, the Bureau had to produce its own pure sucrose (ordinary sugar) and dextrose (corn sugar). However, the manufacturing processes described in German patents were so obscure that reconstruction of the sugars required almost complete original research. NBS was more than successful in its efforts to develop a low cost method for producing almost chemically pure low cost dextrose. In so doing, the Bureau actually launched a new domestic industry.

Between World Wars I and II, Standard Sample activities grew slowly but steadily. Industry requests for materials for chemical analyses were still the major impetus but new Standard Sample needs began to also arise. World War II saw some significant new challenges for NBS. As a primary national laboratory for materials research, NBS contributed to the Manhattan Project through uranium studies. Among many other accomplishments, NBS scientists carried out pioneering work in the separation of uranium isotopes. After the war, NBS received support from the U.S. Atomic Energy Commission (AEC) to develop SRMs for determining the assay and isotopic compositions of uranium and plutonium materials. These certified reference materials are still available today from the U.S. Department of Energy (DOE).

Breakthroughs in the fields of nuclear physics, electronics, and polymer research in the years immediately after the war brought about new demands for Standard Samples. By 1951, there were Standard Samples for spectrographic as well as chemical analysis of raw materials (primarily metals, ores, chemicals, and ceramics) and special hydrocarbon blends for calorimetry. Standard Samples were then developed for the evaluation of color and fading characteristic of materials and for certification of important physical properties such as pH, melting point, and radioactivity.

The 1950s and 1960s saw an even greater acceleration of efforts by NBS scientists and engineers to certify reference materials for physical or special engineering properties and to transfer production of SRMs^8^ to private organizations whenever possible. For example, it was during this period that the extensive NBS hydrocarbon blend project was transferred to the American Petroleum Institute (API). Increased efforts by NBS to expand activities in new, essential areas also signaled the beginning of a change in the nature and scope of NBS Standard Sample activities.

#### 3.3.2 SRMs Come into Their Own

In the early 1960s, NBS realized that Standard Samples would always be important to industry and that industrial demand would continue to grow. It also recognized the contributions Standard Samples could make to help solve measurement problems in such emerging areas of national need as clinical and environmental analysis. Consequently, in 1964, the Office of Standard Reference Materials (OSRM) was established and given direct responsibility for coordinating and directing all Standard Samples activities. Previously, the NBS technical divisions had managed separate components of these activities, coordinating them through the Analytical Chemistry Division. With the establishment of this new office, a number of new program thrusts [6] were identified and initiated, including the start of what was to become a major effort in developing SRMs for clinical chemistry.

NBS and OSRM had its most productive period, in terms of the development of both numbers and types of SRMs, in the decade covering 1969 to 1979. Tremendous growth occurred in such areas as steels and steel making alloys, nonferrous metals and alloys, high purity chemicals, environmental gases, liquids, and solids, ores, ion activity, thermal conductivity, and radioactivity [7]. During the 10 years following (1979–1989), there was an even greater increase in the demand for SRMs in the environmental and clinical areas. Heightened public concerns about the environment and new regulatory requirements were the major driving forces.

The end of the 1980s was marked by several substantive changes for NBS, the institution. In 1988, the U.S. Congress passed the Omnibus Trade and Competitive Act^9^ that changed the NBS to NIST and in so doing, reinforced the newly renamed institution’s mission to support U.S. commerce and trade. As one result of this Congressional action, a new operating unit within NIST, called Technology Services (TS), was established. Concurrently, the OSRM was renamed the SRM Program and placed within OMS, one of the four new directorates comprising TS.

The 1990s can be described as a decade that saw a significant realignment in focus (and physical composition) from SRMs for traditional or classical chemical measurements to SRMs for instrumental measurements and a trend in the development of new SRMs for such “high tech” applications as biotechnology and semiconductors. Increasing requirements for calibration-type measurements by remote sensing devices and real-time, online instrumentation also served to rapidly change the priorities for new SRM development. While 250 SRMs have been discontinued or transferred to other providers in the last 10 years, over 600 new SRMs have been certified to address the new technologies with their own unique certified reference material needs.

### 3.3 Accuracy—The Basis for Measurement Compatibility

**Fig. 4:**
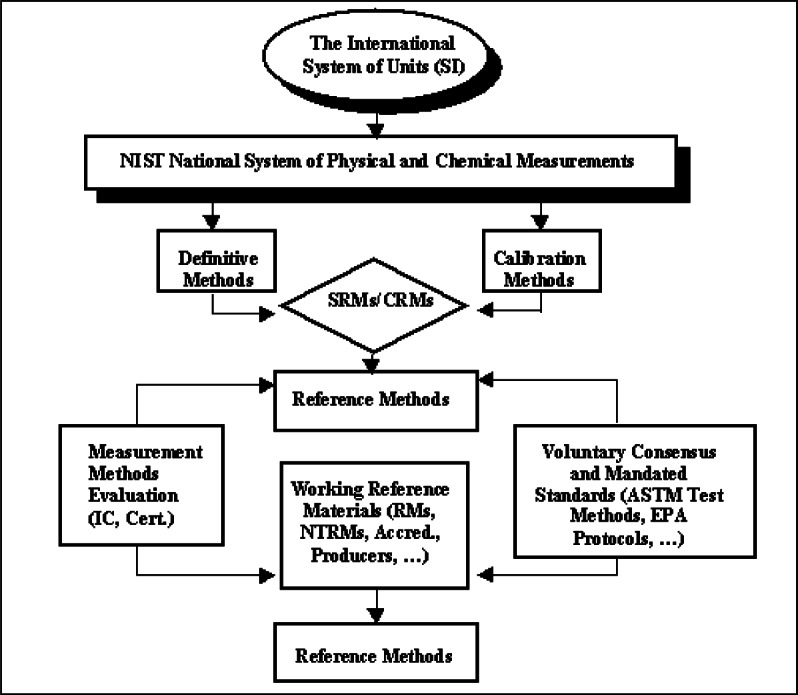
The U.S. National Measurement System for Reference Materials.

NIST SRMs serve as one mechanism for achieving measurement compatibility based on accuracy (see Fig. 4). Compatibility means that the data “agree” and therefore can be compared, regardless of where or when the measurements are taken or what techniques are used. In other words, measurements are compatible when tests for a specific property of a material yield identical values or results, within agreed limits (uncertainties) [8]. Many analysts attempt to base compatibility on precision alone, that is, through the demonstrated repeatability of data (within-laboratory precision) and reproducibility of data (between-laboratory precision). However, in such cases, the relationship between the test value and the true value of a property of a material is not known. Accuracy, the correlation between the measured value and the true value of a property, must be used to obtain data compatibility either among a variety of methods or over a long period of time or both. Combined with a “systems approach” to measurement, SRMs are an important key to achieving accuracy and compatibility.

### 3.4 Producing and Certifying an SRM

It is commonly thought that NIST (and previously, NBS) manufactures as well as certifies all SRMs. For a time after World War II, the Bureau did have several experimental “factories” in operation where the materials for many SRMs were actually produced on site. However, such “factories” no longer exist. These days, NIST selects the candidate materials it plans to certify, establishes their specifications and related properties that will impact various certification parameters, but leaves the actual production to commercial enterprises.

In addition to coordinating the selection and procurement of the candidate materials, the SRM Program manages the certification processes involved. There are two main aspects of SRM certification. First, there are legal ramifications: a NIST SRM carries with it the full weight and authority of NIST and the U.S. Department of Commerce (DOC). Furthermore, many SRMs are specifically authorized by legislation and in recent years, have been mandated by various Federal regulations, particularly in the areas of environmental control. For example, the U.S. Environmental Protection Agency (EPA) requires the use of NIST analyzed gas SRMs in a pollution control program that involves a nationwide network of State and local monitoring stations. The second aspect of SRM certification concerns the more traditional technical requirements that are central to the quality assurance of measurements. These requirements include determination of material homogeneity, material stability, and the assignment of values of properties for which the material is certified.

A NIST SRM is certified on the basis of accuracy; that is, each certified value is the present best estimate of the “true” value and it is not expected to deviate from the “true” value by more than the uncertainty stated on the certificate. This uncertainty includes all estimates of possible systematic error, method imprecision, and material inhomogeneity. Three principal measurement modes are used by NIST for certification of chemical composition SRMs. In order of preference they are:

1. Measurement by a primary or “definitive” method of known and demonstrated accuracy and having negligible systematic errors.
2. Measurement by two or more highly reliable independent methods, or designated reference methods, having uncertainties (including possible systematic errors) that are small relative to the required certification accuracy.
3. Measurement by NIST and a qualified group of cooperating laboratories, generally using definitive or reference methods or both and previously issued SRMs (if available) as controls. This mode is frequently used for renewal of previously issued SRMs or when a method-dependent certification is involved.

NIST generally utilizes modes 1 and 2 to certify new materials but for renewals, mode 3 has proved useful in certifying over 100 metal and metal alloy SRMs, the latter having been accomplished through a 25-year-old cooperative program with the American Society for Testing and Materials (ASTM)^10^. Currently over 50 other SRMs are at various stages of production under the auspices of the ASTM/NIST cooperative research program^11^ and requests for new SRMs of various types and numbers continue unabated [9]. While many reference materials are offered by the entire community of producers around the world, hundreds of new SRMs are still requested every year by U.S. customers alone.

### 3.5 The SRM-Industry Partnership

NBS/NIST has been a leader in reference materials for over 95 years, but it has never worked alone. U.S. industry was its first, and remains to this day its major, partner. Commercial and industrial laboratories and associations, scientific societies, and private standards-making bodies guide and support many SRM activities. Under the ASTM umbrella for example, over 250 different companies are currently cooperating in the certification activities of SRM metals, petroleum, and glasses by providing resources (funds, personnel, materials, or services). Many other organizations and universities also cooperate directly with the SRM Program and the NIST Laboratories to develop and certify SRMs and this partnership continues to expand. Within the clinical, environmental, health, and agricultural areas particularly, a number of SRM projects are underway to better define the health and well being of the population of the United States. Other nations too, have begun to respond to growing demands for standards in support of international trade, world health, and the burgeoning economic systems of third world countries. This, in turn, has spurred global demand for and cooperation in certified reference materials activities on the international level. By law and tradition, NIST is a partner with its sister NMIs in addressing such international needs.

### 3.6 Needs and Goals

As has been described, the NIST SRM Program has diverse and far-reaching responsibilities. To meet these responsibilities in the 21st century and beyond, the SRM Program must analyze measurement trends, establish appropriate priorities, and help focus NIST resources toward the most important needs for new SRMs. Such demands will have to be accommodated while maintaining a balanced program and a continuity of supply of existing SRM services. It is estimated that 200 new SRM types will be required to satisfy domestic needs alone over the next five years and international demand for new certified reference materials will number in the thousands by the end of the decade. Obviously NIST can only address a fraction of these total requirements. Therefore, one of the highest NIST priorities is to cooperate with domestic and international partners so that NIST can leverage its SRMs effectively and in concert with certified reference materials produced elsewhere in the world.

On the global level, NIST believes that there are two requisites to success through cooperation: better information transfer and better technology transfer. Groups such as the International Organization for Standardization (ISO) and the International Union of Pure and Applied Chemistry (IUPAC) must continue to (1) collect information on the availability of certified reference materials, (2) promulgate general guidelines in the areas of terminology, certification criteria, and contents of reference materials certificates and, (3) disseminate this information widely. In the area of technology transfer, greater technical cooperation based on accepted international criteria must be encouraged among the reference materials producers throughout the world in order to expedite production, certification, and distribution. To satisfy as many demands as possible, the SRM Program has reinforced its cooperative activities with ASTM and the NMIs of other countries and instituted more effective needs assessment mechanisms for identifying and evaluating the impact of major areas of new SRM needs.

### 3.8 SRMs in the Future

The current SRM inventory profile is given in Table 1. There are six major technical categories covering some 36 types or classes of SRMs. Maintenance of these standards is critical to the United States but in the future, NIST expects the greatest demands for SRMs will come in two major areas: health/clinical/foods and physical properties. Standards for health care, particularly for diagnostic and treatment purposes, are sorely needed. In 1997, the Standards Committee of the American Association for Clinical Chemistry (AACC) identified the measurement of a particular heart protein, troponin 1 (cTnl), as an important concern for clinical laboratories. This protein is released into the blood when a heart attack occurs and thus is one of the important diagnostic markers for such an event. However, different immunoassays of the protein currently provide vastly different results in the same specimen. NIST has agreed to provide a well-characterized material that will be used as a standard for clinical diagnostic tests for cTnl in human serum plasma.

The Nutrition Labeling and Education Act of 1990 requires that information be provided on product packaging for selected nutrients in all processed foods. To help support this regulatory requirement, NIST is developing a series of food-based SRMs. These standards are intended primarily for the validation of analytical methods used for the measurement of nutrients of foods with similar fat, protein, and carbohydrate composition by processed food manufacturers and commercial and federal food testing laboratories.

The demands for development of SRMs for physical properties and engineering have recently begun to outpace the environmental demands that have for two decades dominated the SRM activities at NIST. Evolving national and international traceability requirements have become the driving force for new SRMs in the areas of telecommunications, semiconductor manufacture, pharmaceutical production, forensic and military applications, remote sensing and environmental monitoring, mechanical properties, and many other areas. The need for SRMs will grow in direct proportion to the development of new technologies and new efforts to improve the quality of life for all people. NBS successfully met its first challenge in 1904 with the issuance of the first Standard Sample and the NIST SRM Program will carry on that proud tradition in the years to come. The SRMP Web site at http://www.nist.gov/srm provides an up-to-date listing of currently available NIST SRMs and RMs.

## 4. Standard Reference Data Program

The results of quantitative scientific measurements are scientific data, the numbers and other information that document our observations, experiments, and calculations. The generation, quality, and dissemination of scientific data have been a prominent central mission of NIST and its predecessor, NBS, for almost its entire century of existence. As a national laboratory dedicated to the advancement of measurement science and engineering, NBS/NIST recognized early in its history that the assessment of the quality of measurement results is a fundamental component of its mission. The following section will review NBS/NIST activities in scientific data, define what is meant by data evaluation, provide an historical perspective, discuss the changes brought by computerization, and set out some future challenges.

### 4.1 Why NIST and Data

The impact of collections of high quality data such as those currently produced by NIST are primarily felt by data users, most of whom are not knowledgeable about the quality of measured data, even if they can find them among the hundreds of thousands of journal articles published each year. Users who are designing new products and processes or who are planning new research and development routinely need quality data for reliable decisions. NIST data collections summarize previous measurement experience and data evaluation assesses the quality of current measurement technology. Both data quality and accessibility are critical to industrial and other data users, and the NIST data programs are an integral part of the measurement services and technology that NIST provides.

### 4.2 Data Evaluation

The primary defining feature of NIST data work is critical evaluation, that is, assessing the quality of reported data. The NIST data programs today are unique in the international scientific community and serve scientists, engineers, and the general public worldwide. The data collections that result from NIST data evaluation efforts are widely distributed and form the largest such collection available.

What is data evaluation? It is the careful examination of measurement results by experts. When evaluating data, three viewpoints are important:

1. Has the data generator identified all the independent variables important to the measurement? Have all these variables been controlled? How has this been documented?
2. How do the data follow the known laws of nature? How do they follow empirical laws?
3. How do the data compare to other measurements that look at the same phenomena?

The mixture of these three viewpoints depends on the maturity of the scientific area and the status of previous evaluations. If the body of data being evaluated is in a new area, or the physical laws are not well known and duplicate measurements do not exist, the emphasis then is put on the first viewpoint. Do we know all the variables involved, and do we document our control of them well enough? For more mature scientific areas, especially when earlier compilations exist, the emphasis gets placed on the latter two viewpoints. The result of data evaluation is a collection of data with quality indicators that allow users to use the data more confidently. An additional result is that the evaluation provides a snapshot of the present-day quality of measurement technology.

The interaction between data evaluation and research on measurement science and technology as executed by NIST is subtle, but real. While the evaluation process results in quality judgments on existing measurement results, an additional benefit is feedback to the data generation community in the form of critiques of experimental and calculational procedures. In this way, the measurement process is refined and improved, and the quality of available data increases. Over the years, such critiques by NIST experts have resulted in great improvements in measurement technology. In a related manner, the expertise of NIST measurement experts is usually far broader than found in other institutions, thereby enabling more valid comparisons among different measurement technologies. NIST experts also maintain a neutrality that is important in making objective evaluations. As the volume of scientific results increases dramatically and the proliferation of measurement techniques grows, NIST data programs continue to serve science and technology by improving the accessibility of data of known quality.

### 4.3 A Brief History of NIST Data Work

The compilation and critical evaluation of property data as an important NBS function can be dated to the Bureau’s involvement in preparation of the *International Critical Tables* [10] in the 1920s. NBS played a leadership role in the project to produce this seven volume series, whose contributors included hundreds of scientists from all parts of the world. The Editor-in-Chief was Edward W. Washburn, Chief of the NBS Chemistry Division and the Editorial Board included NBS Director George W. Burgess. Many Bureau staff members contributed to the work, which is still used and cited 80 years later.

Over the ensuing decades, NBS started new specialized data activities in areas such as phase equilibria for ceramics (1930s), chemical thermodynamics (also 1930s), and atomic spectroscopy (late 1940s). However, the rapid build-up of government supported science and engineering after World War II brought increased demands for a systematic program, fully integrated into the NBS research agenda. NBS leaders such as Allen V. Astin, Edward L. Brady, and Lewis M. Branscomb worked with Congress and the Johnson Administration to establish the world’s first formal government-endorsed data evaluation program. This effort culminated in the enactment of the Standard Reference Data Act of 1968.^12^ This act of Congress established the National Standard Reference Data System (NSRDS), a coordinated and comprehensive program with an objective to “ensure that reliable reference data are easily accessible by scientists, engineers and the general public.” NBS was given the responsibility for coordination of the NSRDS, but other Federal agencies and private organizations were expected to participate. In response, NBS set up a series of formal data evaluation centers that covered a wide range of physical, chemical, and materials disciplines. Joint projects were started with other agencies, professional societies, trade associations, and foreign laboratories. Several publication channels were established, in particular the *Journal of Physical and Chemical Reference Data*, that was published in partnership with the American Institute of Physics (AIP) and the American Chemical Society (ACS).

By the early 1980s, the framework for the present day data program was well in place—a dedication to critical evaluation and quality assessment; the use of computers to the fullest extent possible; and, the need for partnerships whenever practicable. This formula still works today in ensuring the highest possible impact for NIST’s data investments. David R. Lide, Chief of the Office of Standard Reference Data during this period, summarized the program in a classic article in 1980 [11]. It was also during this decade that NBS initiated and executed several large-scale partnerships to address major data needs in both focused and general scientific areas. NIST partnered with the National Science Foundation (NSF) and the U.S. DOE to operate a joint program in data evaluation. The primary purpose was to involve outside expertise in data evaluation work and as a result, well over 100 new projects were funded and completed through a competitive grants program.

NBS had several major programs with professional societies that provided industry with important sets of critically evaluated data. Working with the American Institute of Chemical Engineers (AICE), NBS helped set up the Design Institute for Physical Property Relations (DIPPR). This industry-led consortium has produced high quality, physical property data on several thousand chemical compounds of highest industrial importance. NBS and the American Society for Metals (now ASM International) jointly operated the Alloy Phase Diagram Program that resulted in the definitive collection of binary and ternary alloy phase diagrams. NBS/NIST and the American Ceramic Society continue to execute a similar program for ceramic phase diagrams. NBS and the National Association of Corrosion Engineers (NACE) performed a decade long program to produce evaluated databases and expert systems utilizing high quality corrosion data. All of these programs featured joint NBS-private sector funding and involved international experts.

In the 1990s, the NIST data programs continued to emphasize their unique dedication to data evaluation. Table 2 describes the new programs that were initiated to address data needs in more applied and engineering areas. At the same time, NIST spent considerable effort to use computerization, to the fullest extent possible, in order to make the data programs more efficient and to disseminate the program results.

### 4.4 Major NIST Data Programs

Some of the most important and successful NBS/NIST programs in the area of standard reference data are listed in Table 2. While this brief listing cannot do justice to the many high quality data collections that have been produced by NBS and NIST data experts over the last 50 years, it does demonstrate the breadth and depth of NIST data work. A complete summary of the publications, databases, and online data systems resulting from NBS/NIST data programs is available at http://www.nist.gov/srd.

### 4.5 The Computerized Data Revolution

The widespread availability of computers, the personal computer (PC) explosion, the development of telecommunication networks and the growth of the Internet/World Wide Web have irrevocably changed the generation, collection and dissemination of scientific data. As this Information Revolution became a reality, NBS/NIST took vigorous steps to maintain its leadership in scientific data. With the advent of modern computers, it was natural for NBS data experts to explore how computers could be used both for internal data management activities and for the public dissemination of NBS data collections.

During the 1970s, the Office of Standard Reference Data (OSRD) and the various data centers that it coordinated, embarked on a number of projects aimed at utilizing the growing power of digital computers to improve the efficiency and effectiveness of the NBS data programs. NBS quickly became recognized as a pioneer in this area. The strategy encompassed two paradigms for computer delivery of data that are still used today: installation on one’s own computer and access via networking to remote data collections. In the early 1980s, the first recognizable PCs were just beginning to appear. The essential features of scientific databases stored on PCs are local control, heavy use, inclusion of search software, and the facility to transfer data to computational software and other applications. Online data services are those in which the user connects directly to a remote computer—20 years ago through dial-up telecommunications and in the year 2000, through the World Wide Web.

Throughout the 1980s, every NBS/NIST data activity created databases of references containing the data of importance to their areas of responsibility. Many centers developed specialized data entry programs that captured not only bibliographic information but also the numeric tabular and graphical data contained therein. In addition, the data centers developed suites of software that supported data evaluation through the use of discipline-specific analysis, statistical procedures, and correlation techniques.

Many of the data handling software packages developed at NBS/NIST were used by outside organizations. The NIST Crystal Data Center developed AIDS 80, a powerful package that evaluated and managed crystallographic data. The NIST Alloy Phase Diagram Data Center created a suite of graphical digitization and database management tools that supported the international alloy phase diagram program run jointly by the American Society of Metals and NBS/NIST. A similar set of graphics software for handling ceramics phase diagrams was developed under the NBS-American Ceramics Society Phase Diagram for Ceramists Program.

The computerized dissemination of NBS collections of evaluated data proceeded likewise. In the late 1970s, NBS worked with the U.S. EPA and the National Institutes of Health (NIH) to create and operate the Chemical Information System (CIS), the first online system to provide scientific numerical data. CIS featured a powerful substructure and nomenclature search system that allowed users to search for data on a specific substance and to identify classes of chemical compounds with particular structural features. CIS-integrated databases were built by many groups, including NBS thermochemistry and crystallographic data centers.

At the same time, NBS began offering magnetic tapes of formatted data files suitable for outside users to load onto their own mainframe computers. It was the responsibility of the users to build their own search software and to manage the data. The PC revolution of the 1980s changed all that, and OSRD quickly began offering files on floppy disk similar to those on magnetic tapes. It soon realized, however, that users wanted self-contained packages that were easy to install and that included built-in user interfaces. By 1985, two systems were under advanced development. Steven E. Stein, then of the NBS Center for Chemical Physics, was building a MS-DOS mass spectral data system while Charles Wagner of Surfex^13^ and John R. Rumble, Jr., of NBS were building an x-ray photoelectron spectroscopy (XPS) database on an Apple platform. The mass spectral database was released in 1987 and became an immediate success. Today it is incorporated into virtually every mass spectrometer sold. The next 2 years saw many other NIST databases released including the XPS database.

The blossoming of NIST computerized data dissemination continued unabated into the early 1990s when two approximately concurrent changes hit the computer world: the release of the Microsoft Windows operating system and the Internet explosion. In one short period, the NIST PC databases built for the MS-DOS operating system and proprietary online systems such as the MPD Network became obsolete as users demanded the Windows version of existing MS-DOS data products. Almost at the same time, the Internet and especially the World Wide Web revolutionized online data delivery. Whereas previous online systems required years of development, putting data on the Web now required only several months of work. At the present time, NIST operates 15 Web-based data systems that receive thousands of users every day. The SRDP Web site at http://www.nist.gov/srd contains the most current list of online systems.

### 4.6 Future of Standard Reference Data Activities at NIST

The challenge of producing new and important compilations of critically evaluated data continue to face NIST data activities as the new century begins. Twenty-first century science and technology will take on new characteristics:

1. Research will undoubtedly rely more and more on modeling and simulation.
2. Development of new processes and products will be based on computer-aided design and testing.
3. Quality and model-based processing and manufacturing will drive industry.
4. Large-scale instruments such as the Next Generation Space Telescope will generate copious quantities of data.
5. New laboratory instruments will routinely make higher quality measurements.
6. Quantum chemical codes will routinely calculate properties of arbitrarily large chemical compounds.

All of these developments are related to the future demands for higher quality and greater accessibility of scientific and technical data. What steps is NIST taking to address these challenges?

#### 4.6.1 Data for Applied Science and Engineering

NIST continues to expand its data evaluation activities into new areas. As more complex and realistic applied science and engineering systems are being studied and modeled, NIST is actively meeting the need for quality data. For example, NIST has started work to assess the quality of data on the fire properties of materials and objects as such data are indispensable for reliable fire modeling results. NIST is also expanding its data work to include large molecules and solid-state materials by starting a collaboration with FIZ Karlsruhe (Germany) to update and maintain the inorganic crystal structure database. Likewise, NIST is partnering with Rutgers University and the University of California San Diego to modernize and operate the Protein Data Bank. Recently, a newly released collection of high quality thermophysical data for insulation materials has been evaluated and made Web-accessible.

NIST has also been addressing the need for reliable data collections to support software development, applied mathematics, and statistical analysis. NIST has prepared a large number of validated databases for identification of fingerprints, handwritten and hand-printed materials, and photographs intended to test software that recognizes these objects. NIST also has made available well-characterized data sets for validating statistical software. New data evaluation efforts such as these demonstrate NIST’s recognition that quality assessment of all types of scientific and technical data are critical in today’s age of modeling and simulation.

#### 4.6.2 NIST Data Collections and the Web

NIST and SRDP are taking advantage of the Internet and the World Wide Web to maximize the accessibility of its data collections. Since 1995, NIST has actively built and enhanced a number of Web-based online data systems in every area of its data work. Today the NIST Chemistry WebBook, the NIST Physical Reference Data System, and the NIST Ceramics WebBook are accessed thousands of times each month by all kinds of users seeking the latest NIST reference data. In addition, NIST is working with such partners as IUPAC to make other important reference collections, such as the IUPAC solubility data series, available on the Web.

During 2000, the *Journal of Physical and Chemical Reference Data* became available as full text online. NIST and AIP, today’s joint publishers of the *Journal*, intend to publish in the near future, an online, fully searchable numerical database of the property values. The *Journal* remains the premier publication for evaluated chemical and physical data in the world, and the move to online versions reflects NIST’s dedication in using the latest dissemination technologies. All these activities are all part of the goal of SRDP to make all NIST data Web accessible.

#### 4.6.3 Facilitating Data Exchange, Sharing, and Interoperability

During the 1980s and 1990s, SRDP staff and the Data Centers were instrumental in the development of data exchange standards developed under the auspices of ISO, ASTM, and other groups. These standards were aimed at developing unique data recording formats that could support data dictionary development, database building, and data exchange. In turn, these formats led to more comprehensive standards addressing interoperability issues. Today’s Web environment has increased the need for such standards, with users wanting to share and combine data from the many Web-based data resources that now exist. NIST will continue to provide national and international leadership in the development of scientifically-oriented interoperability data standards.

#### 4.6.4 Linking Computational Science and Databases

It has quickly become clear to NIST that the numbers of chemical compounds, engineering materials, engineering systems, and other objects for which data are needed are growing rapidly. It is equally clear that the compilation of all physical measurements of all proper ties for all compounds, materials, and systems is not possible, or even desirable. Instead, advanced computation and the linkage of computational science to database technology offer hope that through first principles or empirical calculations, needed properties can be obtained. NIST is beginning to explore how such computations would be performed and how the resulting data would be evaluated.

### 4.7 Summary

As NIST moves into its second century and science and engineering moves into the 21st century, the need (and the demand) for better access to high quality data continues to grow. The seven NIST Laboratories, working with SRDP, continue to make data activities a key element of their metrology portfolio. The Information Revolution has touched all areas of science and technology, and data activities are no exception. The challenges facing NIST require the continued dedication and excellence of NIST data experts, working together with their colleagues throughout the world. SRDP is confident NIST can maintain its data leadership in the international scientific community as it meets the future and the new challenges it will bring.

## 5. Weights and Measures Program

### 5.1 An Introduction to Legal Metrology

What is legal metrology? Legal metrology consists of the measurements—and whatever else is related to such measurements (accuracy, records, methods of measurement)—that are established by law or regulation in order to—

- Assure the fairness and equity of commercial transactions, or
- Carry out some other governmental mandate, such as maintenance of health or safety, for its citizenry.

In the United States, the term “legal metrology” is generally synonymous with “weights^14^ and measures” and is usually only applied to the legal requirements governing commercial trade, that is, the buying and selling of goods and services, even though the term has broader meaning. “Weights and measures” in the United States include the regulation of sales by weight or other measure, direct sales over scales (such as in supermarkets, shipping services, or at agricultural exchanges), through pumps (such as at gas stations or dairies), or in sales of pre-measured products (such as packaged food or drugs).

#### 5.1.1 A Very Short History

**Fig. 5:**
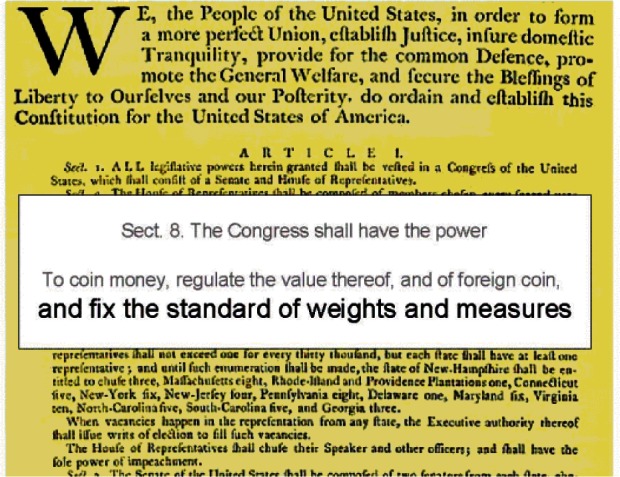
Excerpt from the U.S. Constitution.

Article 1. of the U.S. Constitution gives Congress the authority “to fix” the standards of weights and measures (see Fig. 5). Based on studies conducted by Ferdinand Rudolph Hassler in 1830 that showed no two customhouses in the nation used the same standards of mass or volume, Congress established a national Office of Weights and Measures under Hassler in 1836. This office’s primary purpose was to provide standards of mass, length, and volume to the custom-houses and the States, so that uniform standards would be available for trade purposes throughout the United States. This Office of Weights and Measures became part of NBS when it was founded in 1901 [13]. The Office of Weights and Measures (OWM) continued until renamed the Weights and Measures Program (W&MP) in the 1980s. W&MP continues to operate as the Office of Weights and Measures in the view of all its external customers and stakeholders to the present day.

### 5.2 The State Laboratories

Beginning in the 1830s, the Federal government provided measurement standard artifacts and instruments to the States (another set was provided in the 1870s through the 1890s). However, it was not until 1965 when NBS was again charged with providing the States with standards that a network of State laboratories was established and maintained [14]. The success of this latest program may be attributed to the fact that each State is required to establish a suitable facility and retain permanent laboratory staff trained by NIST in the use of the standards in order to receive new standards and instruments.

**Fig. 6:**
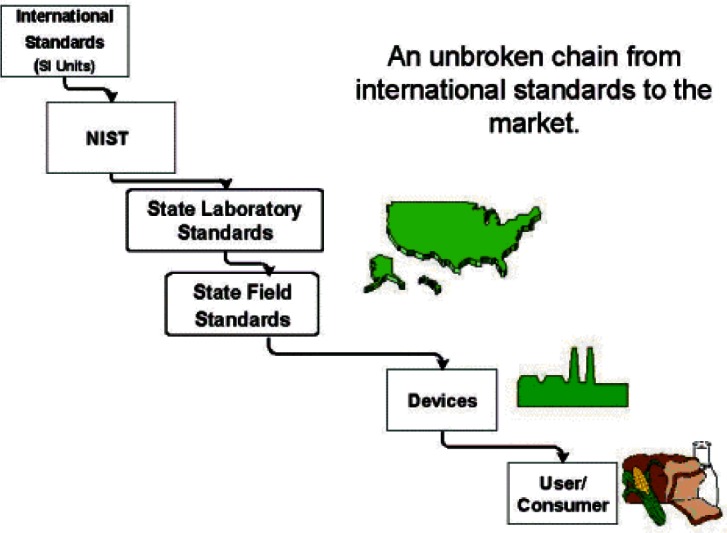
Measurement traceability chain.

The State metrology laboratories now form the backbone of the legal metrology measurement infrastructure in the U.S. (see Fig. 6). The primary method used to achieve equity in the marketplace is a system of traceability from NIST to State laboratories and from the State laboratories to field enforcement staff and registered service agents. NIST provides technical support to the State laboratories through several levels of training, regional measurement assurance programs (RMAPs), and interlaboratory testing all of which are required to meet current recognition requirements. The recognition function of the State Laboratory Program is designed to verify accuracy and traceability in the standards used by local weights and measures field officials.

Each State operates a weights and measures laboratory in mass, length, and volume. Furthermore, although entirely voluntary, most of these weights and measures laboratories also maintain recognition through NIST either in tolerance testing (low level measurement service) or calibration. State weights and measures laboratories have expanded services over the last 10 to 15 years from tolerance testing for legal metrology purposes only, to mass calibration at the highest levels of accuracy in order to respond to the needs of their interstate businesses, local laboratories, and many high-tech manufacturers.

### 5.3 U.S. Legal Metrology is Unique

As a result of exposés on fraud, unsanitary food processing conditions, and adulterated food at the turn of the 20th century, Congress passed reform legislation in significant areas of health and safety, starting with the Pure Food and Drug Act of 1906. Additional legislation was passed that significantly impacted measurements made by private enterprises and government enforcement agencies. However, no national legislation was ever passed to enforce measurement standards for broad scientific, military, industrial development, or trade purposes. There is no national weights and measures law in the United States as there is for virtually every other nation in the world. It may be a fortunate outcome for our capitalistic and entrepreneurial economy that the accuracy and precision of measurements in industrial and scientific applications are left to the needs of the U.S. private sector. Nonetheless, a buyer and seller are not usually equally informed and equally armed with measurement capabilities in a commercial transaction so it has been necessary for the United States to deal with measurement standards for trade in a unique fashion.

### 5.4 Establishment of a Partnership

Measurements made for commercial trade purposes have traditionally been regulated and enforced by State and local governmental agencies, organized, funded, and taking action autonomously one from another. Because of the relative independence of each State with respect to its neighbors, the uniformity of measurement units and their legal requirements has always been a serious issue. It is a tribute to the insight of the first NBS Director, Samuel W. Stratton, that one of his first tasks was to call a meeting of the State weights and measures authorities and weighing industry representatives to discuss the existing and future needs of legal metrology in the United States. It took several years to accomplish, but the first meeting of what became the National Conference of Weights and Measures (NCWM) was held in 1905.

### 5.5 Equity and Uniformity

The priorities established at the first meetings of the NCWM have remained the same throughout its history:

- equity between buyer and seller in every commercial transaction, and
- uniformity of standards, both
- documentary standards, such as laws, regulations, and methods of test, and
- artifact standards of measurement, such as mass, length, and volume standards used by the States and those trading goods and services in the marketplace.

Uniformity of standards provides the most basic market infrastructure for fair competition between businesses as well as for consumer protection.

The States immediately recognized the need for uniform laws and regulations and the first “model weights and measures law” was offered to the NCWM by NBS as early as 1907 and first adopted by a State (New Jersey) in 1911. There are now 10 uniform laws and regulations maintained by the NCWM. Additionally, the first version of what is now Handbook 44, “Specifications, Tolerances, and Other Technical Requirements for Weighing and Measuring Devices,” was published as Bureau of Standards Circular No. 61 in 1917. The first handbook used nationally to check the net contents of prepackaged goods was published in 1959. The current Handbook 133, “Checking the Net Contents of Packaged Goods.” is the national standard for weights and measures officials for package checking.

Weights and measures regulations are aimed at maintaining equity in the marketplace so that

- businesses can compete fairly, and
- buyers and sellers can make informed decisions in trade.

State and local weights and measures activities are designed to assure the accuracy of the measurement upon which the price of the product or service is based. This applies in sales when purchasing groceries, gasoline, heating fuel, or construction materials; when shipping packages, parking in front of a parking meter, or using a laundromat. Weights and measures regulations also cover such varied commercial practices as the sale or purchase of such bulk products as grain or milk by farmers, road construction materials by governments, or building materials by tradesmen and businesses. Weights and measures regulations do **not** cover the sale of such commodities as water, electricity, and natural gas that are controlled by quasi-public self-policing utilities.

### 5.6 Economic Impact in the Marketplace

The lack of accuracy in any measurement system has an associated cost; for example, in a manufacturing process, production of parts that are out of tolerance must often be scrapped. Legal metrology is no different; however, the cost is borne either by the consumer or by business. If the business discovers errors before the product is shipped and subsequently has to rework the product, then the cost accrues to the business (and perhaps if competition allows it, ultimately passes this cost on to the consumer). But if the business does not discover the weights or measures error or chooses to pass the error in product or service on to the consumer, then the consumer bears the cost.

A specific example of the economic impact of legal metrology enforcement in the State of Washington was collected as part of a study to evaluate the minimum level of enforcement needed by a State regulatory agency. The study evaluated 62 large capacity scales (>5000 lb) over which $3.6 billion was sold by weight. Eleven scales were rejected for errors in accuracy (both plus and minus) affecting $465 million of the total product sold by weight. The total economic impact was $30 million. The entire weights and measures program cost only $1 million at the time of the study. One can multiply the Washington State experience to estimate the cost benefit of weights and measures programs across the nation.

**Fig. 7:**
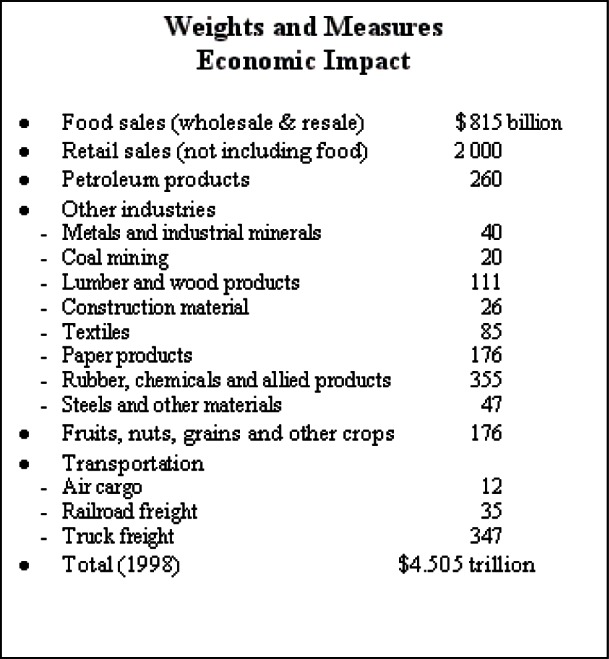
Weights and measures economic impact.

Table 3 shows data from another survey conducted around the same time as the Washington study that found many State programs are only funded between $1 million and $3 million per year, which translates into an annual cost of between 15 cents and 70 cents per person. Yet weights and measures regulations impact 54 % of the $8 trillion U.S. gross domestic product (see Fig. 7).

### 5.7 The Role NIST Plays in This Economic Leverage

**Fig. 8:**
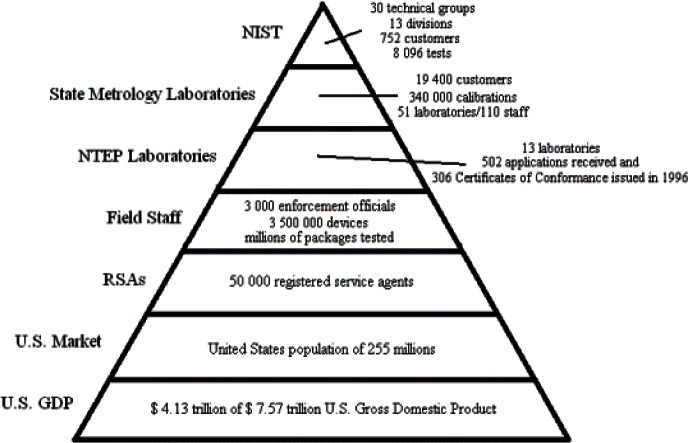
NIST and economic leverage [15].

NIST performs about 8000 calibrations annually for about 800 customers. About 10 to 30 of these calibrations provide direct measurement transfer to the States. Fifty-one State weights and measures laboratories (with a total of 100 staff members) provide 350 000 calibrations for nearly 20 000 customers of which only 51 % are weights and measures regulatory in nature; the remainder supports local, national, and multinational industry needs on a cost-reimbursable basis. The State weights and measures laboratories provide support to about 3000 enforcement officials and 50 000 registered private service agents. Altogether, this system ensures the accuracy of about 3.5 million devices and hundreds of millions of packages produced annually. Fig. 8 best depicts the extent to which NIST calibration services benefit the U.S. each year.

### 5.8 Getting the Public Aroused About a Mundane Issue

NIST (and its predecessor NBS) have never had any regulatory authority over measurements made for trade. Its role has always been “cooperation with the States in securing uniformity in weights and measures laws and methods of inspection.” However, from the start, the NBS role was to provide actual data as to the current state of equity in different areas of trade and to energize its State and local partners to adopt and enforce legal metrology standards. Between 1909 and 1911, NBS staff tested more than 30 000 scales, weights, dry, and liquid measures in more than 3000 stores across the United States. NBS found that

- almost 50 % of the scales,
- 20 % of the weights,
- 50 % of the dry measures, and
- 25 % of the liquid measures

were in significant error in favor of the storekeepers. The annual loss to the consumer in butter alone amounted to more than $8 million. As a result of these findings, many States and cities organized weights and measures agencies, adopted laws and ordinances, and began testing commercial measuring equipment and enforcing trade practices. In 1913, based on an NBS proposal, Congress passed an amendment to the Pure Food and Drug Act requiring the labeling of the net weight, measure, or numerical count of contents on sealed packages of food.

In 1913, NBS began to test railway scales, finding between 75 % and 80 % of the scales unfit for use, some short weighing as much as 3 % to 4 %. Similar or worse results were found in 1917, when NBS tested scales used to weigh coal in mines (and upon which miners’ wages were based). In 1936, NBS tested vehicle and truck scales and showed that it was necessary for States to acquire the capability to enforce weight restrictions for safe highway transport. Slowly and steadily, State and local government agencies have adopted weights and measures laws and regulations patterned after the models provided by NBS to the NCWM.

In the late 1950s, NIST staff again tested scales and gas pumps in the State of Arkansas, which at that time had no weights and measures authority. Huge shortages were found prompting the Arkansas legislature to establish and fund a weights and measures agency. Most recently, in 1997 and 1998, NIST W&MP staff assisted a collaboration of 40 States, the U.S. Department of Agriculture (USDA), the U.S. Food and Drug Administration (FDA), and the Federal Trade Commission (FTC) in the investigation of retail and school prepackaged milk. After widespread notoriety in 1997 of a 45 % short fill (valued at nearly $30 million), the States went back to check milk in 1998 and found less than 20 % of the milk was short filled (valued at slightly over $10 million!). Even though NIST is a non-regulatory Federal agency, it has had the courage to report instances of inequity and help business and government to work in a partnership to correct the situation.

It was not until 1970 that the 48 States adopted some version of the NBS handbook for commercial weighing and measuring equipment. Nonetheless, a general uniformity of standards and practices across the nation had been achieved by the partnership between NBS and the volunteer officers of the NCWM back in their home jurisdictions. As a measure of this successful partnership, Congress has never seen the need for a comprehensive national weights and measures law.

### 5.9 What is the National Conference of Weights and Measures (NCWM)?

The NCWM has evolved from an annual meeting to an ongoing year-round organization with volunteer contributions from State and local weights and measures regulatory officials, measuring device manufacturers, food and consumer packaging industries, Federal officials, and consumer advocates. It is an independent organization that maintains close ties to NIST. The National Conference on Weights and Measures, Inc., incorporated in 1998, is a national professional organization that develops consensus standards in such areas as weighing and measuring device regulation, commodity regulation, motor fuel quality, and administration of regulatory weights and measures programs. Although NCWM standards have no official regulatory authority, they are recommended for adoption as codes and regulations by Federal, State, and local jurisdictions and so are perceived as quasi-regulatory in nature. The Conference currently operates the National Type Evaluation Program (NTEP), initiated by NBS in 1978, to evaluate new commercial measuring device designs against national performance standards [16]. NIST continues to support the NCWM, providing technical advisors to its committees, work groups, technical sectors, and to NTEP. NIST publishes Handbook 44 “Specifications, Tolerances, and Other Technical Requirements for Weighing and Measuring Devices,” Handbook 130, “Uniform Laws and Regulations,” and Handbook 133, “Checking the Net Contents of Packaged Goods.” Training modules based on these handbooks are available from the NCWM, which coordinates training and certification programs for weights and measures officials and training for industry personnel across the nation.

#### 5.9.1 Uniformity of Regulations and Enforcement

While mechanisms are in place to promote measurement uniformity in commercial transactions throughout the United States, it is not always possible to achieve uniformity because adoption by the States of consensus standards developed through the NCWM is voluntary, not mandatory. Thus, different editions of NIST Handbook 44 are in effect from one State to another. Only 16 States have any kind of requirement that packages of perishable commodities must display on the package (and explain the meaning of) a date so that retailers can avoid offering, and consumers can avoid purchasing, potentially spoiled products. Luckily many industries are national in scope producing products or services for the entire nation, rather than for just a single State. Thus, if several States require labeling of the dates by which perishable products must be sold or used, companies packaging for national distribution will provide this information for all States. However, what looks like a national uniformity in requirements is actually national brands driving the competitive marketplace.

### 5.10 Recurrent Measurement Themes

A number of themes have played a part in weights and measures history in the United States. In the never-ending goal to seek uniformity and equity in the marketplace, or put another way, to see the standards fixed in the entire system from national standards down to the business and consumer level, many strategies have been used.

- First, fixing the standards for weights and measures requires more than just documenting a definition or providing standards to U.S. customhouses and the States. Because of the unique nature and multitude of weights and measures enforcement groups, recalibration services, technical assistance and training, guidance on the use of standards, and standardized regulations and procedures have all played a part in securing a fixing of the standards used in commerce.
- Second, because of the unique nature of the system, collaboration, partnerships, and leveraging of resources have played a crucial part in maintaining an efficient and effective system of weights and measures in the United States. The combined system is greater than the sum of its parts through collaborations with Federal, State, and local governments, and industry. The alternative, used in many other countries, is a national weights and measures program with a national law. The U.S. approach to State and local weights and measures administration and enforcement is particularly effective considering the size of the country, the regional variation in the agricultural and industrial base, the differences in State and regional approaches to consumer protection, and the differences in political considerations in the conduct of the programs. However, some of these factors also can contribute to the lack of uniformity in weights and measures programs and practices across the country.
- Third, many studies have been mentioned throughout this section. These studies were coordinated at the national level and numerous instances were found where problems existed. These nationally coordinated studies seek to measure the degree of accuracy in the marketplace and then continuously improve those areas where problems exist. Again the primary goal is to achieve uniformity and equity in the marketplace.
- Lastly, W&MP has always sought to coordinate information on a national level and then to transfer that information and knowledge throughout the entire system. This has been done through the
  - development of uniform regulations that are adopted in the many enforcement areas;
  - development, publication, and wide dissemination of specifications and tolerances for standards and measuring devices;
  - development of and training in the procedures for calibrating standards and verification of measuring devices and practices;
  - development of statistically sound procedures for sampling and checking the net content of packages; and
  - training of administrative, laboratory, and field staff in State and local weights and measures programs.

### 5.11 Future Directions for W&MP

Information technology and the Internet have permitted pilot efforts by W&MP in ways to disseminate information and deliver education in timely, efficient, and effective ways. For example, the program operates a “fax-on-demand” system that allows anyone with a telephone and fax to request and then retrieve a wealth of information from NIST without having to worry about W&MP office hours or if a particular staff person is available. The W&MP Web site (http://www.nist.gov/owm) has already proven extremely popular with weights and measures stakeholders nationally and internationally. In the near future, it will be expanded into a searchable site that contains much more historical and technical information than are currently available.

Another promising approach for collaboration, partnerships, and education is being used in the State Laboratory Program. Laboratories with PCs linked by network and modem are being equipped with teleconferencing cameras, microphone headsets, and off-the-shelf software that allows them to communicate over the Web visually and verbally in real time in the laboratory. It is also possible to exchange methods and answer questions anywhere in the system with from 2 to 40 laboratories hooked together and communicating at the same time. Soon CD-ROM based technology will be used as supplemental training in basic laboratory metrology.

#### 5.11.1 Quantity and Quality Control

To support modern commerce, rapidly and highly accurate measurements are critical. Companies seek greater efficiencies by controlling quantity and quality variables using new measuring devices, measurement technologies, and measuring features that, in turn, increase demands on the commercial measurement system. However, product developers in small- and medium-sized businesses often lack access to technical criteria and the knowledge of weights and measures regulations in order to design measuring instruments or packages correctly the first time. This can lead to product delays and lost profits. Therefore, one objective of W&MP is to make a wide range of technical information and training material accessible to industry and weights and measures officials at the product design, quality control, and enforcement levels.

#### 5.11.2 E-Commerce

Conducting business over the Internet offers new opportunities and challenges. Weights and measures laws and regulations apply to this form of business in the same manner that they apply to the “brick and mortar” businesses, but the methods of presenting information and techniques most suitable to consumers buying habits are different. Additionally, information available to a consumer in a store where visual comparisons of products are available to the consumer may not be available on-line. Consequently, development of the appropriate policies that will enable businesses to take advantage of the Web while ensuring that consumers still have the ability to make value comparisons and informed decisions is a major W&MP project.

### 5.19 International Role

Although NBS/NIST has had a long history of providing a national perspective in a regulatory environment largely enforced at the local level, the W&MP role of bridging local and international perspectives will become increasingly important in the future. Globalization of the marketplace has occurred and the corresponding reduction of tariffs has increased the significance of normative standards, labeling requirements, and product certification requirements as technical barriers to trade. To address this issue, NIST began in the 1980s to bring its neighbor Canada into NCWM discussions, culminating in reciprocal prototype evaluations for certain commercial devices. Facilitating trade in all of the Americas with respect to legal metrology issues is now a primary NIST objective. But this will require increased educational efforts to influence other nations on why the United States prefers that industry and consumers benefit from legal metrology requirements based on performance rather than design specifications. W&MP has partnered with the NIST Technical Standards Activities Program, which represents the United States in the International Organization of Legal Metrology (OIML), and the NIST Global Standards Program to provide representation and insight on the unique U.S. perspective in weights and measures standards negotiations abroad. As the global economy becomes a significant portion of the U.S. domestic economic well being, NIST and W&MP have to play a greater role than ever before in promoting U.S. concepts and uniformity in global trading standards and methods.

### 5.20 Summary and Challenges

While the W&MP collective goal is to seek equity and uniformity in the marketplace, how to most efficiently and effectively accomplish that as a country is a challenge. We must constantly question the prioritiyulyulzation of resources and assignment of tasks to obtain the greatest impact. No government program has ever had an overabundance of resources; thus, good stewardship calls for effective prioritization. Evaluating how W&MP can best provide technical leadership and guidance at the national level requires finding ways to leverage NIST efforts and resources to gain the support and buy-in of others who will effectively implement practices on the national and international levels. This requires cooperation, collaboration, and partnership, especially since the national coordination of local efforts is not backed with funding for implementation at the local level. NIST must constantly be on the lookout for new partnerships with organizations and people who share NIST’s vision of its mission.

The U.S. legal metrology system has evolved for more than two hundred years to reach its current level of uniformity. The system has many voluntary and privatized elements about it, but one thing has never changed—its goal to seek and preserve the U.S. democratic process and respect States rights in the adoption and application of documentary standards for weights and measures. NIST/OMS and W&MP are committed to making this unique system the most effective weights and measures system in the world.

## Figures and Tables

**Table 1:** SRM^®^ Technical Categories

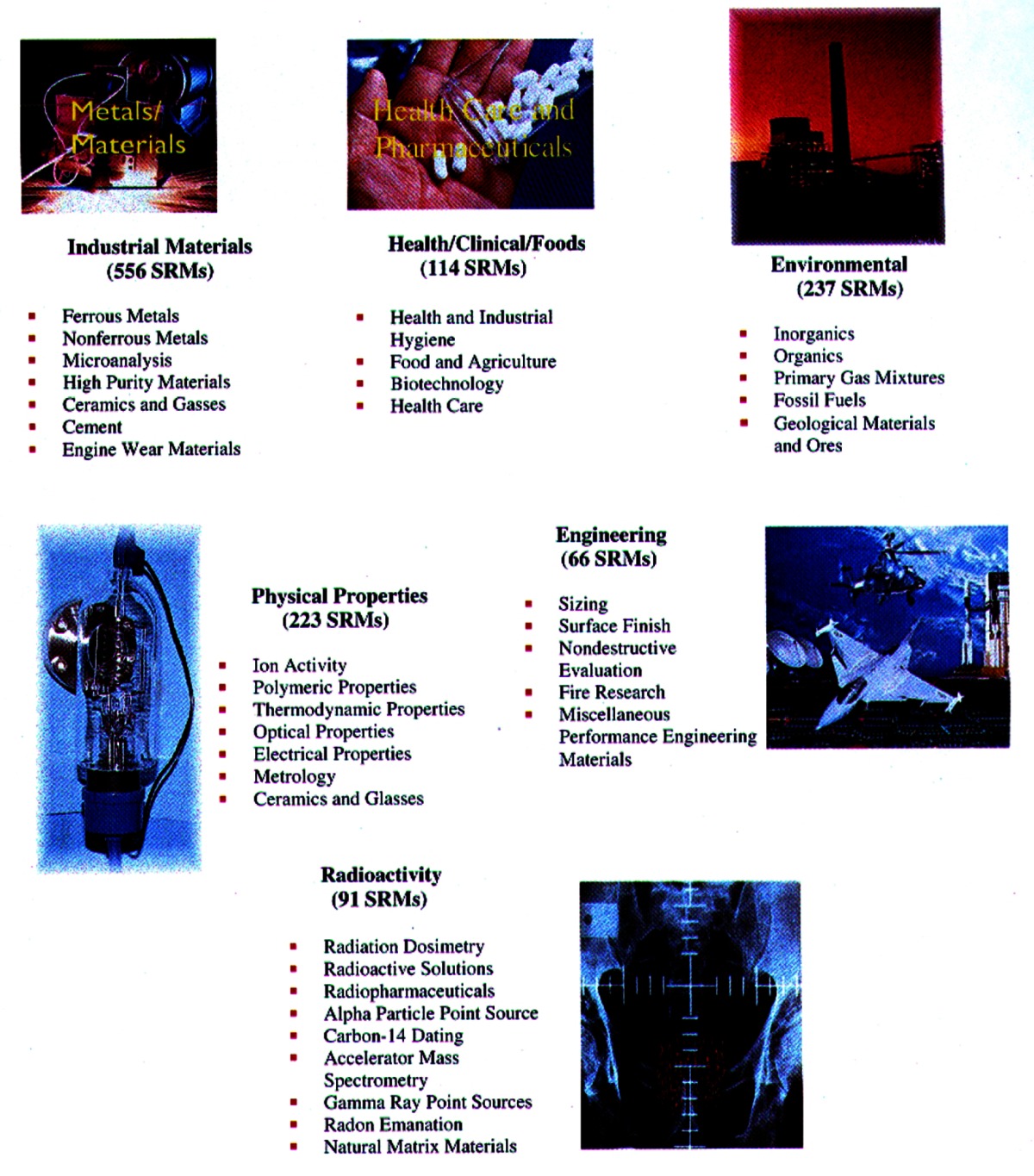

**Table 2:** Overview of some NBS and NIST Data Programs, 1970–2000

**Physics**	
Fundamental constants	A decennial update of the fundamental physical constants.
Atomic and molecular spectroscopy	Electronic, vibrational, and rotation energy levels and transition probabilities for atoms, molecules, and transient species.
Radiation physics	High energy interactions of electrons and photons, especially of use for health physics.
**Chemistry**	
Chemical thermodynamics	Standard state data for inorganics and small organics; thermochemical tables; energetics of molecular ions; in partnership with the Thermodynamic Research Center at Texas A&M University.
Chemical kinetics	Reaction rates for gas phase reactions in the atmosphere, combustion, and other applications.
Fluid properties	Thermophysical properties and equations of state of industrial fluids, refrigerants, air, water, petrochemicals; in partnership with the Gas Producers Association, NASA and other groups.
X-ray photoelectron spectroscopy	Characterization of the chemical environment of surfaces.
Mass spectrometry	A library of mass spectra produced originally by NIH and EPA and now maintained and fully evaluated by NIST.
Stability constants	Stability constants in solution; in partnership with Texas A&M University.
**Materials**	
Single crystal data	Unit and reduced cells, chemical data for single crystal diffraction; in partnership with the International Centre for Diffraction Data.
Thermal and electrical conductivity	A long term project at Purdue University.
Corrosion data	Corrosion rate and other data; in partnership with the National Association of Corrosion Engineers.
Alloy phase equilibrium	Binary and ternary phase diagrams; in partnership with ASM International.
Ceramics phase diagrams	Phase diagrams for ceramics; in partnership with the American Ceramic Society.
**Information Technology and Mathematics**	
Statistical reference datasets	Well-characterized data sets for analyzing the performance of statistical packages.
Datasets for recognition software	Well-characterized data sets for assessing the performance of software to identify fingerprints, pictures, handwriting, etc.
**Bioinformatics**	
Protein Data Bank	Structure of biomacromolecules; with Rutgers University and University of California at San Diego.
Thermodynamics of enzyme-catalyzed reactions	Thermochemical properties of important biochemical reactions.
**Electronics**	
Plasma modeling for semiconductor manufacture	Collision and reaction data to support modeling of semiconductor manufacture.
**Construction and Fire Science**	
Insulation materials	World’s largest collection of thermal properties of insulating materials.
Fire test data	Properties of materials and objects in fire tests, used for modeling real-life fire situations.

**Table 3:** Annual cost of weights and measures protection^a^

State	Population	W&M total Annual budget $	Annual $ per capita
TX	18 378 000	2 610 855	0.142
IL	11 752 000	3 161 100	0.269
NC	7 070 000	2 960 000	0.419
MD	5 006 000	1 562 110	0.312
MN	4 567 000	2 937 000	0.643
AZ	4 075 000	2 400 000	0.589
KS	2 554 000	1 200 000	0.470
NE	1 623 000	1 100 000	0.678

a Based on 1996 Telephone Survey and Census Data.

## Footnotes

1 NIST Authorization Act, Public Law No. 100-519, SEC. 109.

2 International Organization for Standardization (ISO)/International Electrotechnical Commission (IEC) Guide 25:1990, *General requirements for the competence of calibration and testing laboratories*.

3 ISO/IEC 17025:1999, *General requirements for the competence of calibration and testing laboratories*.

4 ISO 9000 Compendium: 1994, *International standards for quality management*.

5 Trademark was first registered by NIST in 1993.

6 Founded in 1896, the organization was renamed the American Foundrymen’s Society in the 1940s, and on 1 July 2000, became the American Foundry Society (AFS).

7 Standard Sample No. 17, Certificate of Analysis dated February 1, 1906.

8 The term, “Standard Reference Material (SRM)”, was officially put into use by NBS in 1965 and is synonymous with the internationally accepted term “certified reference material (CRM).”

9 Public Law (P.L.) 100–418, August 1988.

10 The ASTM/NBS Memorandum of Agreement on Production of Standard Reference Materials was signed June 21, 1977 and renewed between ASTM and NIST June 1996.

11 ASTM/NBS Research Associate Program Memorandum of Agreement was signed Febuary 2, 1976.

12 Public Law (PL) 90-298, now designated as 15 U.S.C. 290.

13 Certain commercial equipment, instruments, or materials are identified in this paper to foster understanding. Such identification does not imply recommendation or endorsement by the National Institute of Standards and Technology, nor does it imply that the materials or equipment identified are necessarily the best available for the purpose.

14 Traditionally the term “weight” has been used in commercial and everyday parlance as being synonymous for mass. In the context of “weights and measures,” the SI unit of the quantity “weight” is the kilogram (kg)[12].
